# Exposure to Flood Hazards in Miami and Houston: Are Hispanic Immigrants at Greater Risk than Other Social Groups?

**DOI:** 10.3390/ijerph13080775

**Published:** 2016-08-01

**Authors:** Alejandra Maldonado, Timothy W. Collins, Sara E. Grineski, Jayajit Chakraborty

**Affiliations:** Department of Sociology & Anthropology, University of Texas at El Paso, El Paso, TX 79968, USA; amaldonado7@miners.utep.edu (A.M.); segrineski@utep.edu (S.E.G.); jchakraborty@utep.edu (J.C.)

**Keywords:** hazard, environmental justice, vulnerability, flood, Hispanic or Latino, immigrant

## Abstract

Although numerous studies have been conducted on the vulnerability of marginalized groups in the environmental justice (EJ) and hazards fields, analysts have tended to lump people together in broad racial/ethnic categories without regard for substantial within-group heterogeneity. This paper addresses that limitation by examining whether Hispanic immigrants are disproportionately exposed to risks from flood hazards relative to other racial/ethnic groups (including US-born Hispanics), adjusting for relevant covariates. Survey data were collected for 1283 adult householders in the Houston and Miami Metropolitan Statistical Areas (MSAs) and flood risk was estimated using their residential presence/absence within federally-designated 100-year flood zones. Generalized estimating equations (GEE) with binary logistic specifications that adjust for county-level clustering were used to analyze (separately) and compare the Houston (*N* = 546) and Miami (*N* = 560) MSAs in order to clarify determinants of household exposure to flood risk. GEE results in Houston indicate that Hispanic immigrants have the greatest likelihood, and non-Hispanic Whites the least likelihood, of residing in a 100-year flood zone. Miami GEE results contrastingly reveal that non-Hispanic Whites have a significantly greater likelihood of residing in a flood zone when compared to Hispanic immigrants. These divergent results suggest that human-flood hazard relationships have been structured differently between the two MSAs, possibly due to the contrasting role that water-based amenities have played in urbanization within the two study areas. Future EJ research and practice should differentiate between Hispanic subgroups based on nativity status and attend to contextual factors influencing environmental risk disparities.

## 1. Introduction

Hazards and environmental justice (EJ) research reveals that socially marginalized groups are typically highly vulnerable to risks and disasters [1,2,3,4]. This includes people of Hispanic/Latino origin in the US, who have been found to experience disproportionate exposure to hazards, as well as a constrained ability to prepare for and recover from disasters [5,6,7,8,9]. Despite the fact that hazards and EJ studies have examined the social vulnerabilities of Hispanics, almost all have treated the Hispanic population as a single homogeneous group (except for [7,10,11,12,13]). This is problematic because it conceals substantial differences in language, nativity and social class. For example, foreign-born Hispanics (i.e., Hispanic immigrants) may be particularly vulnerable to disasters due to their insecure residency status, lower incomes, and English language deficiencies. However, when all Hispanic people are lumped together in one category, as has been done in most prior analyses, the distinctive characteristics of Hispanic immigrants are concealed. Hispanic immigrants are distinct from US-born Hispanic people and other racial/ethnic groups not only in terms of immigration status but also linguistically and culturally. Disregarding those and other differences leaves a significant gap in hazards and EJ research, as has been pointed out in previous studies [10,11,13]. Due to this gap, hazard reduction efforts may fail to properly serve groups such as Hispanic immigrants. As the Hispanic immigrant population continues to grow in the US [14], it is of increasing practical importance to expand knowledge regarding the sources and consequences of the environmental injustices and hazard vulnerabilities they experience.

Although there is a dearth of hazards and EJ research focused on the disproportionate risks experienced by Hispanic immigrants, research shows that floods are a significant threat to humans. Due to growing populations and assets invested in coastal cities, societal exposures to flood hazards are increasing throughout the world [15,16]. Over 50% of the US population lives in coastal zones, with this proportion projected to increase [17]. Furthermore, due to climate change and sea level rise, flood exposure is generally increasing, even without accounting for demographic shifts [18,19]. This is especially true along the US eastern seaboard and Gulf Coast regions, which have seen high rates of sea level rise [20]. Given the heightening risks, it is important to enhance our knowledge of human exposure to flooding, especially for the purposes of better protecting those who are at greatest risk to these hazards.

In order to provide an understanding of EJ issues specific to Hispanic immigrants, we analyze residential flood risk at the household level, with a focus Hispanic immigrants in comparison to other social groups within the Miami and Houston metro areas, both of which are flood prone and contain large Hispanic populations. This analytical approach is novel, as previous studies have used aggregated census data and tended to treat Hispanics/Latinos as a monolithic group. By comparing Hispanic immigrants to other social groups, including US-born Hispanics, our study is designed to clarify whether they experience disproportionate exposure to flood risk, adjusting for other variables known to influence flood risk.

Our primary research question is: Are Hispanic immigrants disproportionately exposed to risks from flood hazards, adjusting for other race/ethnicity/nativity categories, housing tenure, socioeconomic status, flood self-protection, flood risk perception, and the desire to live near water-based amenities? Based on previous hazards research, we hypothesize that Hispanic immigrants will experience disproportionate exposure to flood risks when compared to other social groups, including US-born Hispanics, non-Hispanic Blacks, non-Hispanic other minorities and non-Hispanic Whites. In what follows, we first review the literature on EJ in relation to flooding, as well as studies focused on other variables known to influence flood risk at the household level. We then introduce our study methods, before presenting and discussing analysis results. We conclude by highlighting the scholarly and practical relevance of the study findings.

### 1.1. Race, Ethnicity, SES and Environmental Injustice in Flood Hazard Exposure

Despite flood hazards being framed as an environmental injustice issue by only a small body of literature [21], studies on race/ethnicity and flood exposure have found that minorities may be disproportionately exposed in some contexts [12,22,23,24,25,26], and that the heightened exposure of minorities to hazards often has historically unjust roots. In Austin (TX, USA), periodic flooding was a factor in the racial segregation of the city, where Hispanics and Blacks were marginalized to areas most prone to flooding [27]. Austin has remained racially segregated, with marginalized groups disproportionately exposed to hazards [28]. In metro Miami, non-Hispanic Black and Hispanic populations were found to be significantly more likely to reside within areas exposed to inland flood risks [29]. In New York City, even though minorities were not disproportionately represented within 100-year flood zones on a city-wide basis, they were overrepresented in flood zones of several of the city’s boroughs [30]. In California, Hispanic residents were found to be overrepresented as residents of floodplains when compared to other groups [31]. In terms of the consequences of residential flood exposures, a lack of US citizenship and Hispanic ethnicity were associated with significantly worse health outcomes among people in homes impacted by a flood disaster in El Paso, TX, USA [32]. Other studies have not found that minority groups experience disproportionate exposure to flood hazards. Some studies, in fact, have yielded contradictory findings. For example, in metro Miami, it was found that non-Hispanic Whites were overrepresented, while non-Hispanic Blacks and Hispanic people were underrepresented, as residents of areas at risk to coastal flooding [29]. Due to these divergent findings, it is important that research takes contextual factors into consideration when examining flood risk disparities between racial/ethnic groups.

In terms of socioeconomic status (SES), numerous post-event studies of flood disasters reveal that low SES groups tend to experience greater vulnerability. However, distributive EJ studies of pre-event relationships between social characteristics and spatial exposures to flood risks have produced less conclusive results. Post-event, low SES can increase vulnerability to flood-related challenges, as has been found to be the case in multiple studies across different contexts [33,34]. Lower SES reflects poverty, lower educational attainment, and livelihood insecurity, and is often associated with renter home occupancy and poorly constructed or maintained housing, as well as reduced capacities to mitigate hazards [1,24,26,30]. For example, Burton and Cutter [35] found that in the California counties of San Joaquin, Sacramento and Yolo, areas with low SES were disproportionately exposed to flood risks associated with failure-susceptible levees. Despite such studies documenting that those of lower SES are typically constrained in their capacities to prepare for, respond to and recover from floods and other hazards [36,37,38,39,40,41,42], distributional EJ studies focused instead on pre-event relationships between SES and flood hazard exposure have yielded ambiguous findings. For instance, in the US and UK, several studies have found that in certain contexts, socially-advantaged individuals may experience the greatest pre-event flood exposure [13,29,43,44]. Taken together, these findings suggest that SES must be accounted for when examining relationships between racial/ethnic status and flood risks.

### 1.2. Water-Based Amenities and Flood Hazards

Water-based amenities are often correlated with heightened exposure to flood hazards, since such amenities and flood risks are not easily divisible from one another (or easy to separate from one another, as both are natural features of proximity to bodies of water) [25,45,46,47]. Thus, living at risk to flooding may be driven in part by corresponding locational environmental benefits [1]. For example, economists have found that properties located within the 100-year coastal flood zone on North Carolina’s Outer Banks had higher cash values relative to similar properties outside of coastal flood zones [48]. In fact, some of the most expensive real estate in the US is located in areas at high risk to flooding [49]. This means that flooding can devastate even wealthy predominantly White waterfront communities that enjoy the benefits of access to coastal amenities, as was the case in some affluent areas of New Orleans during Hurricane Katrina [2]. Research at the neighborhood level in metro Miami suggests that racial/ethnic minority groups tend be overrepresented as residents in areas exposed to inland flood risks with fewer water-related amenities [6,29], while residents who are economically advantaged and non-Hispanic White are overrepresented in amenity-rich coastal areas at risk to flooding [6]. Although advantaged groups may inhabit areas at high risk due to the associated locational benefits, they also typically have greater capacity to mitigate the risks associated with residential flooding [1,50]. For instance, wealthier communities are able to reduce flood risks through self-protective actions such as making (expensive) home modifications, maintaining flood insurance to fully compensate for property damages or losses, and by exercising their collective social power to receive improved community flood protections from government sources (e.g., flood walls, levees, beach nourishment programs, etc.) [1,6]. Since the pursuit of water-based amenities by economically affluent people may confound relationships between social variables of interest and flood exposure, it is necessary account for the effects of water-based amenities in EJ analyses of flood hazard exposure.

### 1.3. Self-Protection from Flood Hazards

Self-protection strategies allow residents to defend themselves from the devastating impacts of flooding. In the context of flood hazards, self-protection can include structural changes to the home and nonstructural actions. Structural mitigation actions include elevating and flood-proofing homes, both of which are effective means of mitigating flood losses [51,52,53,54,55]. Elevation involves raising the home so that the lowest floor and critical infrastructure systems are above the flood level. Elevation can reduce flood losses to near zero. Studies have found that flood-proofing can reduce flood losses by 21% to 89%, depending on whether wet proofing (i.e., when portions of the home are allowed to flood) and/or dry-proofing (i.e., when actions are taken to prevent entrance and enable removal of floodwaters) have been implemented [53,54,55].

In terms of nonstructural self-protection strategies, in the US, flood insurance plays an important protective role, since it provides compensation for property losses due to flooding. In 100-year flood zones {i.e., designated by the US Federal Emergency Management Agency (FEMA) as “Special Flood Hazard Areas”}, where there is at least a 25% chance of flooding during a 30-year mortgage period, flood insurance is required in homes and buildings with mortgages from federally-regulated or insured lenders. Coverage may be obtained through private insurers, but the vast majority of flood insurance policies in the US are acquired through the National Flood Insurance Program (NFIP), which is administered by the FEMA. While mortgage holders or owners (unlike non-owners or renters) are responsible for maintaining flood insurance for home structures, any household—no matter whether they rent the home, are paying on the home, or own the home outright—may purchase flood insurance coverage for the home’s contents through the NFIP. Because these structural and nonstructural self-protection strategies can measurably mitigate or compensate for the impacts of flood hazards, they are important to control for when analyzing the relationship between social variables and exposure to flood risks.

### 1.4. Risk Perceptions and Flood Hazards

Although multiple factors influence individuals’ risk perceptions (e.g., age, ethnicity and previous experiences with natural hazards), risk perceptions may be significantly related to proximity to a hazard [56,57,58]. For example, Heitz et al. [59], Kellens et al. [60] and Lindell and Hwang [61] found that higher levels of risk perception were associated with residents’ locations in flood zones. Furthermore, it has been found that individuals’ residential proximity to flood hazards may influence their self-protective intentions and behaviors [58,62]. Thus, adjusting for flood risk perception is necessary in order to clarify relationships between social variables of EJ interest and flood risk exposure, since a householder’s perception of flood risk may shape their decision-making in manner that leads them to select a residence at lower vs. higher flood risk.

## 2. Materials and Methods 

### 2.1. Study Areas

In addition to being home to more than 900,000 Hispanic immigrants, the Houston-Sugar land-Baytown (Houston) and Miami-Fort Lauderdale-West Palm Beach (Miami) Metropolitan Statistical Areas (MSAs) face high levels of exposure to flood risk. With a total population of just under 6.5 million residents, the Houston MSA is the sixth largest in the US. According to the ACS 2014 1-year estimates, Non-Hispanic Whites comprise 38% of the Houston MSA population, followed by Hispanics (36%), and non-Hispanic Blacks (17%). Hispanic immigrants comprise 14% of the Houston MSA population. Hurricanes and flooding have posed serious and recurring problems in Houston and more than US $112 billion in property assets are covered under the NFIP [63]. According to the National Weather Service, Houston has been hit with hurricanes and flooding that have led to deaths as well as billions of dollars in damage. In April 2016, a major flood event left 7 dead, damaged thousands of homes, and caused at least $5 billion in damage [64]. In May 2015, the Houston area was affected with flooding that damaged thousands of homes and left at least 4 dead and many more displaced [65]. Other events include Tropical Storm Allison (2001), which killed 22 people, damaged thousands of homes, and caused widespread flooding; Hurricane Rita (2005), which was responsible for damages due to strong winds and disastrous storm surge flooding that led to 7 deaths and an estimated $10 billion in damage; and Hurricane Ike (2008), which caused 28 deaths and more than $1 billion in property damage.

**Figure 1:**
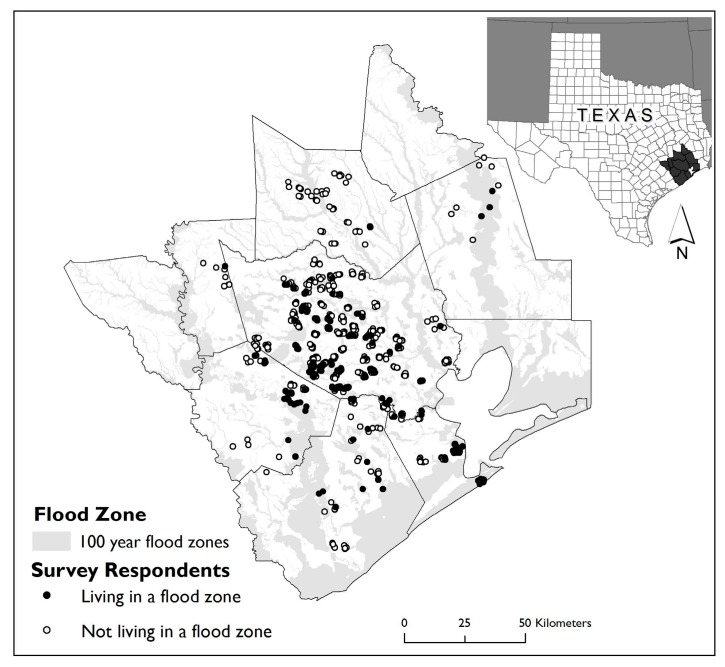
Flood zones and approximate locations of survey respondents in the Houston MSA, Texas study area.

**Figure 2:**
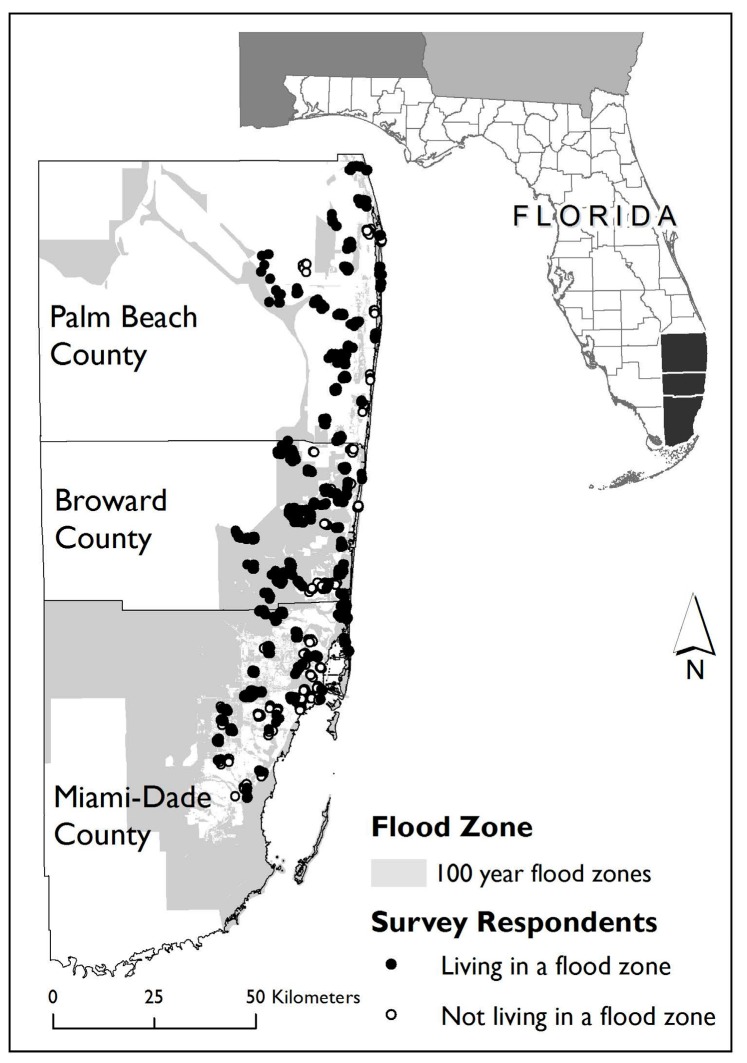
Flood zones and approximate locations of survey respondents in the Miami MSA, Florida study area.

With a total population of over 5.9 million (ACS 2014 1-year estimates), the Miami MSA is the seventh largest in the US. Hispanics comprise 43% of the population, followed by non-Hispanic Whites (32%) and non-Hispanic Blacks (20%). Hispanic immigrants comprise 26% of the population. A study of coastal flood risk ranked Miami first in asset exposure and fourth in population exposure for cities worldwide [66]. Over US $212 billion in property assets are covered under the NFIP [63]. Because of its location between the Gulf of Mexico and the Atlantic Ocean, Miami is one of the most hurricane-prone urban areas in the world. Hurricane Andrew provides an example of the devastation experienced in Miami due to hurricanes. Andrew struck southern Florida and south-central Louisiana in 1992 and was the costliest natural disaster in US history at the time with damage estimated at nearly US $25 billion. Hurricane Andrew hit Miami-Dade County especially hard, resulting in at least 15 deaths and leaving up to one-quarter million individuals homeless [67]. Figure 1 and Figure 2 depict the geographic contexts of the two study areas, including flood zone boundaries and the approximate home locations of the study participants.

### 2.2. Data Collection

An institutional review board (IRB)-approved telephone survey was administered among 1283 randomly selected adults living in the Houston and Miami MSAs from June through July 2012. The human subjects research protocol (FWA #: 00001224; internal IRB reference #: 261207-4) was approved by the University of Texas at El Paso IRB on 18 May 2012. Survey participants were selected using probability-based methods and a two-stage sampling strategy to obtain a sample that was socially and spatially representative of the MSAs [68,69]. The two-stage sampling strategy implemented for each MSA consisted of the following. First, quadrants containing the same number of tracts in each MSA were defined, and within each quadrant, census tracts were stratified into quintiles based on percent non-Hispanic White and median household income, which were created from US census data. Within each quintile (of each quadrant) 6 census tracts were randomly selected, for a total of 30 census tracts per quadrant. Within each of the 120 selected census tracts in each MSA, phone-based structured surveys were then completed with at least 5 randomly selected householders. The goal was to complete 600 householder surveys within each MSA. Here, we analyze data for 546 householders from the Houston MSA and 560 from the Miami MSA for whom we had complete data for the majority of analysis variables (derived from the survey). The survey had a response rate of 33%, which is comparable to that achieved in recent published studies based on random digit dialing surveys [70]. Most SES and demographic survey items were derived from the American Community Survey instrument (version 2011). The survey was conducted in English and Spanish. It was written in English, and then subjected to three translation iterations, including a back translation. The telephone interviews were conducted by trained, English–Spanish bilingual interviewers employed by a firm with expertise in survey research with Hispanic populations. Incentives of $10 in cash were provided to all survey participants. All responding householders verbally consented to participate and were 18 years of age or older.

### 2.3. Independent Variables

#### 2.3.1. Race/Ethnicity/Nativity

The study employs categorical race/ethnicity measures, which were constructed by re-coding self-identified data from householders on ethnic status, racial status and, for Hispanic participants, nativity status in order to define the groups. The following categorical measures of race/ethnicity/nativity are employed in analyses: 1 for “Foreign-born Hispanic” (Hispanic respondents born outside of the US) and 0 for not; 1 for “US-born Hispanic” (Hispanic respondents born inside the US) and 0 for not; 1 for non-Hispanic “Black” (Black/African American respondents who were not Hispanic) and 0 for not; 1 for non-Hispanic “Other Minority” (American Indian/Alaska Native, Asian/Pacific Islander, or “other race” respondents who were also not Hispanic and not Black) and 0 for not; and 1 for non-Hispanic “White” (White only respondents who were not Hispanic) and 0 for not. Since our focus is on whether or not Hispanic immigrants experience disproportionate risks in terms of exposure to flood hazards, we utilize the Hispanic immigrant group as the reference group in our models. Table 1 provides details on the construction of the analysis variables. Table 2 reports descriptive statistics for the analysis variables; for all dichotomous variables, the mean is interpretable as a proportion.

#### 2.3.2. Socioeconomic Status (SES)

Respondents’ SES is analyzed using two variables. First, we use a factor comprised of two variables consisting of educational attainment and household income (Houston Cronbach’s Alpha = 0.609; Miami Cronbach’s Alpha = 0.633). Educational attainment is measured based on a survey question that gauged for the level of education obtained by the individual in the household with the highest level of education. Household income is measured based on response options to a survey question that gauged the total household income of survey respondents in 2011 before taxes. We use renter-occupancy as a second SES indicator, measured by a survey item that determined whether they rented or owned their residences. For this variable, the survey asked householders to indicate if their home was: (i) owned by them or someone in the household with a mortgage of loan; (ii) owned by them or someone in the household free and clear; (iii) rented; or (iv) occupied without payment of rent, but not owned. For our analysis, this variable was coded as *1* for renter (iii) and *0* for owner (i, ii, or iv).

#### 2.3.3. Water-Based Amenities

We measure the role of water-based amenities in residential decision-making among householders using survey items that gauge their preferences when making residential location choices. Two survey measures that represent the degree to which survey respondents were influenced in moving to their current residences by specific considerations are used, which focus on the following features: (i) proximity to the coast or beach; and (ii) proximity to a river or lake. Survey respondents indicated the importance of each of those two features in the choice of their current home using a scale ranging from 1 to 5, with 1 = “not a consideration at all” to 5 = “a very important consideration.”

#### 2.3.4. Self-Protection

##### Structural: Flood Mitigation

The composite variable for flood mitigation is based on yes/no responses to seven survey items that gauge whether protective action against flooding had been taken at respondents’ home sites (Table 1). Responses to each were coded as 1 for “yes” and 0 for “no” and all seven items were summed into one composite variable that ranges from 0 to 7, indicating how many flood mitigation actions were implemented at each home site.

##### Non-Structural: Flood Insurance

For the flood insurance measure, a survey question gauged whether or not the contents of respondents’ homes were insured through the National Flood Insurance Program. We focus on maintenance of contents insurance since homeowners and renter-occupants alike are eligible to maintain NFIP contents insurance, whereas only homeowners are eligible to maintain insurance for home structures. “Yes” responses are coded 1 and “no” options as 0.

#### 2.3.5. Flood Risk Perceptions

Four Likert-scale type survey items were used to assess respondents’ perceptions regarding the seriousness of flood problems in their community as well as their levels of concern regarding potential flood impacts upon their households. Table 1 reports coding each of the 4 items used to construct the flood risk perception measure. Responses to these 4 items were applied to create 1 factor for each metro area that was used for our analysis (Houston Cronbach’s alpha = 0.860; Miami Cronbach’s Alpha = 0.873).

### 2.4. Dependent Variable: Residential Exposure to 100-Year Flood Risk

Our dependent variable is a measure of the presence/absence of respondents’ homes in the 100-year flood zone. This variable was derived using householders’ geocoded home locations and FEMA Digital Flood Rate Maps (DFIRMs). This dependent variable measures each respondent’s risk as 1 for within a 100-year flood zone and 0 for outside of 100-year flood zone.

### 2.5. Analytic Strategy

We conducted preliminary bivariate group comparisons to explore differences between our independent variables with respect to flood risk. To do so, we analyzed the relationship between our binary independent variables—which consist of our race/ethnicity/nativity categories, renter status and NFIP contents insurance—and our dependent variable by employing Pearson chi-square tests for differences between presence vs. absence within flood zones. Next, we used Mann–Whitney U tests to analyze relationships between our scale variables—which include our SES, water-based amenities, structural self-protection, and flood risk perception measures—and our dependent variable.

Then, we employed generalized estimating equations (GEEs), a multivariate analysis technique appropriate for dealing with clustered data, in order to analyze determinants of residential flood risk. Prior to modeling GEEs, we applied multiple imputation (MI) to address missing values in the survey data and reduce non-response bias. MI techniques appropriately adjusts the standard errors for missing data [71] and MI is considered a best practice for dealing with missing data in statistical analysis [72,73,74]. We imputed missing values for 20 datasets to ensure that the multi-parameter significance tests for our pooled models were valid [71]. We report pooled GEE results from analyses of all 20 datasets. Data were analyzed by modeling two GEEs (one for each MSA) using the independent variables described above as predictors and the 100-year flood risk measure as the outcome, while accounting for clustering at the county level. The models adjust for clustering based on the county of residence because previous studies of EJ and vulnerability in the context of flood hazards have identified counties as units that strongly influence human-flood hazard relationships in the US [6,75,76,77]. GEEs are an appropriate method of analysis for this study given that they provide a general method of clustered dichotomous variables and relax several assumptions of traditional regression models [68,69]. Binary logistic GEEs were specified, based on the distribution of the dependent variables (binomial), and the working correlation matrix structure was specified as exchangeable, since this assumes constant intracluster dependency [78].

Data for Houston and Miami were analyzed separately, with results for each city examined comparatively to explore contextual differences and similarities. Datasets were created for each of the two study areas, each consisting of identical analysis variables, and the datasets for each city were statistically analyzed in parallel manner. The Hispanic immigrant category was left out of the GEEs, meaning that it serves as the reference group for the other race/ethnicity/nativity categories. Linear binary logistic regression diagnostic tests showed no multicollinearity issues among analysis variables for either study area.

## 3. Results

### 3.1. Bivariate Group Comparisons

Table 3 reports bivariate group comparison results in terms of differences in survey respondents’ characteristics based on their residence within vs. outside 100-year flood zones. Pearson chi-square test results are reported as the percentages of individuals residing inside vs. outside the 100-year flood zone for each of the dichotomous independent variables. Mann-Whitney U test results are reported as mean values for each scale independent variable in terms of households residing within vs. outside 100-year flood zones. In Houston, representation within vs. outside 100-year flood zones is statistically significant for three of the race/ethnicity/nativity groups. Hispanic immigrant and “other minority” households are overrepresented as residents of flood zones (vs. non-Hispanic immigrant and non-“other minority” groups, respectively), while non-Hispanic White households are underrepresented in flood zones. Additionally, mean flood mitigation is significantly higher among households not residing within 100-year flood zones. None of the bivariate relationships between the other independent variables and flood risk are statistically significant.

In Miami, none of the racial/ethnic/nativity groups are significantly over- or under-represented in 100-year flood zones, although the result for non-Hispanic Whites approaches statistical significance for overrepresentation. A significantly higher proportion of households with (as compared to those without) NFIP contents insurance reside in flood zones. Higher mean SES approaches statistical significance in terms of the association with residence in flood zones. None of the bivariate relationships between the other independent variables and flood risk approaches statistical significance in Miami.

### 3.2. Generalized Estimating Equations

Houston GEE results indicate that, adjusting for relevant covariates, US-born Hispanics, non-Hispanic Blacks and non-Hispanic Whites experience significantly lower odds of exposure to 100-year flood risks than do Hispanic immigrants (Table 4). US-born Hispanics, non-Hispanic Blacks, and non-Hispanic Whites are, respectively, 67%, 53%, and 75% less likely than Hispanic immigrants to reside within a 100-year flood zone. Thus, among the comparison groups, non-Hispanic Whites exhibit the lowest likelihood of exposure to flood hazards relative to Hispanic immigrants. The other minority group and SES factor indicate positive relationships with residing within the 100-year flood zone at a statistically non-significant level. Renter-occupancy and proximity to the coast/beach show negative, statistically non-significant associations with the 100-year flood zone. Proximity to river/lake indicates a positive relationship with residence in a 100-year flood zone at a statistically non-significant level. Our results also show that lower levels of home site flood hazard mitigation are significantly associated with greater flood exposure in Houston, adjusting for other variables. A one standard deviation increase in flood mitigation is associated with a 32% decrease in the odds of residing within the 100-year flood zone. Adjusting for other variables, the relationship between having flood insurance for the home’s contents (vs. not) and residing in a flood zone approaches statistical significance (*p* = 0.057) and is positive; having insurance is thus associated with greater odds of residing within the 100-year flood zone. Lastly, lower levels of risk perception are significantly associated with greater flood exposure; a one standard deviation increase in the risk perception factor is associated with 19% reduction in the likelihood of living in a flood zone.

GEE results for Miami indicate statistically significant relationships for being non-Hispanic White, having high scores on the SES factor, and having contents flood insurance with inhabitancy in the 100-year flood zone (Table 4). In Miami, being non-Hispanic White is associated with a 73% greater likelihood of residing in a 100-year flood zone than being Hispanic immigrant. The directionality of the relationships in the GEE indicate that the other racial/ethnic/nativity groups experience lower odds of residential exposure to 100-year flood risks than Hispanic immigrants, although those relationships are statistically non-significant. A one standard deviation increase in SES is significantly associated with 24% greater odds of residing in the 100-year flood zone. Renter-occupancy and both water-based amenities variables exhibit positive, non-significant relationships with the 100-year flood zone. Higher flood mitigation exhibits a negative relationship with the 100-year flood zone, at a statistically non-significant level. Having NFIP contents insurance (compared to not having it) is significantly associated with a 98% greater odds of residing in the 100-year flood zone. Lastly, higher flood risk perception exhibits a negative relationship with 100-year flood risk, with this relationship being statistically non-significant.

## 4. Discussion

In terms of the relationship between race/ethnicity/nativity and flood risk, when comparing the analysis results for Houston and Miami, clear differences emerge. In Houston, results generally align with expectations derived from the EJ literature, since socially marginalized Hispanic immigrants experience significantly greater flood risk than non-Hispanic Whites. Our results also indicate that US-born Hispanics and non-Hispanic Blacks exhibit significantly less flood risk than Hispanic immigrants in Houston, which is a novel finding. However, in Miami, a contradictory pattern emerged in terms of non-Hispanic Whites and 100-year flood risk. In Miami, non-Hispanic Whites exhibited significantly greater odds of exposure to 100-year flood risks than Hispanic immigrants. While US-born Hispanics and non-Hispanic Blacks exhibited lower exposure to 100-year flood risks than Hispanic immigrants in Miami, those results were not statistically significant. The possibility that Hispanic immigrants may be at greater (as in Houston) and lesser (Miami) risk than non-Hispanic Whites has been masked in prior studies, including one which examined Hispanics as a single ethnic group at the census tract level in Houston and Miami [39]. In that study, census tracts in Houston with higher proportions of Hispanics were found to have significantly less 100-year flood risk, while the proportion of tract residents who were Hispanic in Miami was associated with statistically non-significantly greater flood risk [39]. This underscores the importance of disaggregating the Hispanic population into relevant subgroups whenever possible, since doing so allowed us to uncover that it is specifically Hispanic immigrants and not US-born Hispanics that differ from non-Hispanic Whites in terms of their exposure to flood risks.

Higher SES is associated with greater flood exposure in both cities; the association is non-significant in Houston and significant in Miami. In terms of housing tenure, similarities exist between the two MSAs, as the association between renter status and 100-year flood risk is statistically non-significant and positive. The positive associations between higher SES and pre-event flood risk found here aligns with other studies in the US and UK where a similar relationship has been found [13,29,43,44]. While they face increased odds of exposure, people of high SES are not considered to be particularly vulnerable to flooding; for example, they typically possess a greater ability to mitigate against flood hazards [1,24,26,30,34,36,37,38], thus reducing their potential losses during a flood event.

In terms of water-based amenities, both MSAs share similarities in that none of the associations these variables have with exposure to the 100-year flood zone is statistically significant, and the associations were positive. This positive association between rating the proximity to a river or lake as a more important consideration when selecting the current home and greater odds of flood risk is expected based on the literature [25,47,48,49].

The association between having flood insurance for contents of the home and 100-year flood risk is positive and approaches significance in Houston, while this association is positive and significant in Miami. This association was expected, as residents of flood zones are encouraged to maintain flood insurance (e.g., owners with mortgaged homes are required to maintain flood insurance on structures within 100-year flood zones). The statistical non-significance of the relationship in Houston suggests that NFIP contents insurance may be underutilized there, and that public awareness should be increased about the importance of maintaining flood insurance. Less flood mitigation is associated with greater flood exposure in both MSAs, but the association is significant only in Houston. Practically, this points to a need to increase public awareness about the importance of implementing structural flood mitigation measures at home sites, especially in Houston.

Lower flood risk perceptions are associated with greater flood exposure in both Houston and Miami, but the association is statistically significant only in Houston. These results do not align with the literature, as most studies indicate that heightened risk perceptions are associated with closer proximity to flood hazards [60,61,62]. Practically, this suggests that residents of flood zones, especially those in Houston, may tend to underestimate flood risks, and that there is a need for better targeted communication regarding flood risks to residents who reside in flood zones.

From a traditional EJ perspective, our results for Houston generally correspond with expectations, while our results for Miami oppose expectations. Similar to our analysis of Houston, previous studies have found that racial/ethnic minorities and those of relatively low SES experience higher vulnerability to environmental hazards in terms of being unable to take protective action, as well as being adversely impacted by and less able to recover from disasters [12,22,23,24,25,26]. However, in Miami, we found that non-Hispanic Whites and higher SES households were at greater risk to flooding. These results align with those from a previous study of Miami that found significant associations between lower neighborhood-level economic insecurity (or poverty), as well as lower proportions of Blacks relative to Whites, and higher flood risks [39].

Recall that access to water-based amenities and exposure to flooding are often indivisible from one another and thus typically highly correlated. As a result, socially-advantaged groups, such as wealthier and White people, could reside at disproportionately high risk, given that they also are afforded privileged access to amenities, such as the beach. Prior neighborhood-level studies in Miami support that logic, since they have found strong positive relationships for coastal flood risk with high SES, the proportion of non-Hispanic White residents, and area-based measures of amenity values (i.e., mean housing values, the proportion of seasonal/recreational housing units, public beach access) [29]. This suggests that the water-based amenities variables we analyzed, which are based on the level of consideration given to these amenities when the household chose to occupy their current home, do not adequately capture the role of coastal amenities in structuring social relationships with flood hazards in the Miami MSA. In contrast to prior studies [6,29], we employed household-level measures related to the importance of water-based amenities when the householder chose the home, which did not exhibit significant relationships with flood risk in either our bivariate or multivariate analyses. This suggests that relationships between water-based amenities and flood risk in Miami are structured at a coarser scale—indeed, the entire MSA appears to be socio-economically structured by the amenity values associated with proximity to the sandy beaches that line the Atlantic coast—and determined to a lesser degree by preferences for environmental amenities as expressed through household-level decision-making. Thus, a possible avenue for future research is utilizing multi-level modeling in order to develop a better understanding of individual and neighborhood level factors that shape societal patterns of exposure to flood hazards in Miami and elsewhere.

In Miami, socially-advantaged households appear inclined to place themselves at risk to flooding in exchange for residential benefits that come with the risk, under the condition that flood insurance is available to externalize the economic risks of flooding. This is supported by the relationship found between having contents flood insurance and residence in the 100-year flood zone, which indicates that residents of flood zones in the Miami MSA rely on subsidized flood insurance through the NFIP to offset risks. On the other hand, minority groups and those of lower SES in Miami appear to be less exposed to flood hazards due to their financial inability to tap the sought-after amenities that proximity to the coast affords; by extension, our results suggest that minority and low SES residents of the Miami MSA experience environmental injustice based on their constrained access to coastal amenities (see [79], which substantiates this point).

In addition, the demographic composition of the Miami MSA is somewhat unique in comparison to other US MSAs. The Hispanic immigrant population of Miami in particular is highly diverse, being composed of immigrants from many different countries of origin, many of whom have relatively high SES. Because immigration status in Miami is not highly correlated with lower SES, as it is elsewhere in the US, it stands to reason that the pattern found in Miami may differ substantially from other US metro areas.

In contrast to Miami, residential settlement across Houston is far less structured by coastal amenities, despite this MSA being adjacent to the coast. The main economic activities taking place along the coast bounding the Houston MSA are associated with the petrochemical industrial complex, which is among the largest in the world and a major source of air pollution. Such water-based economic activities represent major residential *disamenities*. Put simply, many landscapes at high risk to flooding in Houston have relatively lower water-based amenity value for residents and consequently, they tend to be inhabited by more socially vulnerable people. As was the case in Miami, the two individual-level amenity variables were not statistically significant, suggesting again that relationships between water-based amenities and flood risk are structured at a coarser scale. Thus, the disjuncture between coarse-scale water-based residential amenities and flood risk in the Houston MSA is perhaps the reason why those findings align with traditional EJ expectations. 

Additionally, Houston’s Hispanic population, as mentioned before, differs greatly from that of Miami. First, the Hispanic immigrant population in Houston is much more homogeneous, with more than 80% of immigrants here being of Mexican origin. Second, the Hispanic immigrant population in Houston is generally of relatively low SES, as it is elsewhere in the US. It is thus not surprising that results found here are better aligned with findings from the EJ literature.

## 5. Conclusions

This study examined whether Hispanic immigrants are disproportionately exposed to risks from flood hazards relative to other racial/ethnic groups, adjusting for relevant covariates. In Houston, our analysis indicates that Hispanic immigrant (i.e., foreign-born) status places households at increased flood risk compared to other groups, including Hispanics who are US-born. Flood mitigation and risk perception were found to be significant and negative predictors of flood risk. Factors found to be significant predictors of flood risk in Miami, including non-Hispanic White race/ethnicity, higher SES and NFIP contents insurance, were statistically non-significant in Houston. In Miami, the observed differences suggest that non-Hispanic White and wealthier households experience heightened exposure to flood hazards, but that their risks may be partially offset by high rates of flood insurance coverage.

Our key findings have several important implications. The increased flood risk experienced by Hispanic immigrants in Houston underscores the need for future EJ and hazard vulnerability studies to differentiate more carefully between minority subgroups, particularly within the highly diverse Hispanic population. In this study, nativity status among Hispanics was shown to have an impact on flood risk, but there may be additional factors that amplify vulnerability. For instance, it has been argued that undocumented immigrant status [80] and even country of origin [6] may contribute to the social vulnerability experienced by certain minority groups. Ultimately, distinguishing between subgroups can help pinpoint the characteristics that place individuals at heightened vulnerability.

Recognizing the differences experienced by different Hispanic subgroups may help in developing targeted interventions for reducing their vulnerability. For instance, communities with Hispanic immigrant populations should make disaster information readily available in Spanish. Given that Hispanic immigrants tend to come from collectivist cultural backgrounds, their familial connections may provide more effective channels of disaster communications than other conduits, such as mass media [81]. Familial modes of communication may target specific family members as entry points, for example, children could be provided with bilingual information about flood risks and prevention/recovery resources at school. Public assistance in times of emergency and recovery should also focus on providing aid in a safe space that makes any individual, regardless of immigrant status or cultural differences, comfortable seeking help. Safe spaces can be cultivated through deepened community engagement, culturally-competent approaches, and participatory methods, as well as partnerships among universities, public health agencies and community-based organizations [82].

We found that Miami households of higher SES are at disproportionately high 100-year flood risk. Although wealthier people may be exposed to flood hazards in some contexts, from an EJ perspective it is important to recognize that socially privileged groups have greater capacities to prepare for, respond to, and recover from flood events. This suggests that, when examining flood risks from an EJ perspective, a distinction between hazard exposure and social vulnerability should be made, since they may not always be positively correlated.

The divergent results (between the two MSAs) may be due to distinctions in Hispanic population characteristics and the role of water-based amenities in structuring human-flood hazard relationships. Future studies should adopt a comparative approach focused on multiple study sites, as different contexts may be characterized by different types and levels of water-based amenities that selectively attract particular social groups to live amid flood hazards. As our results demonstrate, different contexts may be characterized by divergent social patterns of exposure to flood risks.

## Figures and Tables

**Table 1:** Variables, survey questions and coding.

Variable	Survey Questions	Coding Used in Analysis
Hispanic Immigrant	(1) Were you born outside the US? (2) Are you of Hispanics, Latino, or Spanish origin?	0 = No 1 = Yes
US-born Hispanic	(1) Were you born outside the US? (2) Are you of Hispanics, Latino, or Spanish origin?	0 = No 1 = Yes
Non-Hispanic Black	(2) Are you of Hispanics, Latino, or Spanish origin? (3) Which of the following best describes your race? Black of African-American	0 = No 1 = Yes
Non-Hispanic Other	(2) Are you of Hispanics, Latino, or Spanish origin? (3) Which of the following best describes your race? American Indian or Alaskan Native, Asian, Pacific Islander, or Some other race	0 = No 1 = Yes
Non-Hispanic White	(2) Are you of Hispanics, Latino, or Spanish origin? (3) Which of the following best describes your race? White	0 = No 1 = Yes
SES Factor		Continuous
Education	Thinking about the person in your household who is 18 years of age or older with the highest educational degree received or level of school completed—what is the highest grade or level of school that this person has completed? 0 = No formal education 21 = Ph.D. degree	
Median Household Income (2011 $US)	What was your total HOUSEHOLD income for the year 2011 before taxes? 1 ≤ $10,000 2 = $10,000–19,999 3 = $20,000–29,999 4 = $30,000–39,999 5 = $40,000–49,999 6 = $50,000–74,999 7 = $75,000–99,999 8 = $100,000–149,999 9 = $150,000–249,999 10 ≥ $249,999	
Housing Tenure	Is this home…? (1) …owned by you or someone in this household with a mortgage or loan—including home equity loans? (2) …owned by you or someone in this household free and clear—without a mortgage or loan? (3) …rented? (4) …occupied without payment of rent, but not owned?	0 = Owner (options 1, 2, 4) 1 = Renter (option 3)
Proximity to Coast or Beach	What level of consideration was given to “Proximity to Coast or Beach” when you constructed, purchased or rented your current home?	1 = Not a consideration at all 5 = A very important consideration
Proximity to River or Lake	What level of consideration was given to “Proximity to River or Lake” when you constructed, purchased or rented your current home?	1 = Not a consideration at all 5 = A very important consideration
Flood Mitigation Composite		0 = no mitigation actions taken 7 = all 7 mitigation actions taken
	Which of the following flood protection methods have been used to protect the home site you occupy from flooding? (1) Home structure elevated to protect against flooding (2) Electric components of the home were elevated (3) Indoor heating, ventilation and air conditioning system components were elevated (4) Outdoor service equipment were elevated (5) Floodwalls, berms or levees were built on site (6) Back flow valves or check valves were installed (7) Interior drainage system was installed	
Flood Insurance (Contents)	Are the contents of the home currently covered by the NFIP?	0 = not covered by NFIP 1 = covered by NFIP
Risk Perception Factor		Continuous
Risk Perception (General)	How much of a problem do you think flooding is in the Metro Area? 1 = “not a problem at all” 5 = “a very serious problem”	
Risk Perception (Property)	How concerned are you about the possibility of a flood causing damage to your home or property? 1 = “not concerned at all” 5 = “extremely concerned”	
Risk Perception (Health)	How concerned are you about the possibility of a flood causing injuries or health problems to you or to members of the household? 1 = “not concerned at all” 5 = “extremely concerned”	
Risk Perception (Livelihood)	How concerned are you about the possibility of a flood preventing your or members of your household from being able to work or causing disruption to daily activities? 1 = “not concerned at all” 5 = “extremely concerned”	
100-Year Flood Risk		0 = outside of a 100-year flood zone 1 = within a 100 year-flood zone

**Table 2:** Descriptive statistics.

Variable	MSA	N	Min	Max	Mean	SD	% Missing
Hispanic Immigrant	Houston	69 (1) 459 (0)	0	1	0.131	N/A	3.297
	Miami	144 (1) 403 (0)	0	1	0.263	N/A	2.321
US-Born Hispanic	Houston	45 (1) 483 (0)	0	1	0.085	N/A	3.297
	Miami	31 (1) 516 (0)	0	1	0.057	N/A	2.321
Non-Hispanic Black	Houston	101 (1) 423 (0)	0	1	0.193	N/A	3.846
	Miami	75 (1) 470 (0)	0	1	0.138	N/A	2.679
Non-Hispanic Other	Houston	18 (1) 500 (0)	0	1	0.035	N/A	5.128
	Miami	10 (1) 524 (0)	0	1	0.019	N/A	4.643
Non-Hispanic White	Houston	298 (1) 227 (0)	0	1	0.568	N/A	3.846
	Miami	289 (1) 257 (0)	0	1	0.529	N/A	2.500
SES Factor	Houston ^1^	N/A	−3.1	2.3	0.000	1.000	N/A
	Miami ^2^	N/A	−2.4	2.1	0.000	1.000	N/A
Education Level	Houston	N/A	1	21	14.831	3.150	1.099
	Miami	N/A	0	21	15.029	3.253	1.071
Household Income	Houston	N/A	1	10	4.766	2.602	23.443
	Miami	N/A	1	10	4.376	2.549	25.536
Housing Tenure	Houston	100 (1) 432 (0)	0	1	0.188	N/A	2.564
	Miami	115 (1) 424 (0)	0	1	0.213	N/A	3.750
Proximity to Coast or Beach	Houston	N/A	1	5	2.449	1.533	9.890
	Miami	N/A	1	5	2.340	1.481	8.036
Proximity to River or Lake	Houston	N/A	1	5	2.072	1.473	10.440
	Miami	N/A	1	5	2.183	1.483	8.036
Flood Mitigation Composite	Houston	N/A	0	7	3.610	1.585	43.407
	Miami	N/A	0	7	3.974	1.604	45.000
Home Elevated	Houston	227 (1) 211 (0)	0	1	0.518	N/A	19.780
	Miami	219 (1) 240 (0)	0	1	0.477	N/A	18.036
Home Electric Components Elevated	Houston	339 (1) 114 (0)	0	1	0.748	N/A	17.033
	Miami	361 (1) 115 (0)	0	1	0.758	N/A	15.000
Home Ventilation System Elevated	Houston	448 (1) 83 (0)	0	1	0.844	N/A	2.747
	Miami	474 (1) 70 (0)	0	1	0.871	N/A	2.857
Outdoor Service Equipment Elevated	Houston	322 (1) 204 (0)	0	1	0.612	N/A	3.663
	Miami	408 (1) 121 (0)	0	1	0.771	N/A	5.536
Floodwalls, Berms, or Levees Installed	Houston	162 (1) 349 (0)	0	1	0.317	N/A	6.410
	Miami	251 (1) 258 (0)	0	1	0.493	N/A	9.107
Backflow Valves Or Check Vales Installed	Houston	185 (1) 200 (0)	0	1	0.481	N/A	29.487
	Miami	183 (1) 216 (0)	0	1	0.459	N/A	28.750
Interior Drainage System Installed	Houston	72 (1) 364 (0)	0	1	0.165	N/A	20.147
	Miami	73 (1) 372 (0)	0	1	0.164	N/A	20.536
NFIP Contents Insurance	Houston	243 (1) 273 (0)	0	1	0.470	N/A	5.495
	Miami	257 (1) 253 (0)	0	1	0.504	N/A	8.929
Risk Perception Factor	Houston ^3^	N/A	−1.8	1.7	0.000	1.000	N/A
	Miami ^4^	N/A	−1.8	1.6	0.000	1.000	N/A
How Much of a Problem is Flooding	Houston	N/A	1	5	3.660	1.239	2.015
	Miami	N/A	1	5	3.766	1.136	0.893
Property Damage	Houston	N/A	1	5	2.782	1.393	0.000
	Miami	N/A	1	5	2.791	1.433	0.179
Health Problems	Houston	N/A	1	5	2.730	1.392	0.183
	Miami	N/A	1	5	2.810	1.418	0.536
Disruption to Daily Activities	Houston	N/A	1	5	2.954	1.426	0.549
	Miami	N/A	1	5	3.018	1.408	0.357
100-Year Flood Risk	Houston	74 (1) 472 (0)	0	1	0.136	N/A	0.000
	Miami	293 (1) 267 (0)	0	1	0.523	N/A	0.000

^1^ Cronbach’s alpha = 0.609; ^2^ Cronbach’s alpha = 0.633; ^3^ Cronbach’s alpha = 0.860; ^4^ Cronbach’s alpha = 0.873; Notes: Means for the dichotomous indicators are presented because they can be interpreted as the proportion of the respondents in the category coded as 1. For example, the mean for Hispanic immigrant in Houston is 0.131, which means that 13.1% of respondents in Houston are Hispanic immigrants.

**Table 3:** Group comparisons by presence/absence in 100-year flood zone.

Variable	Houston			Miami		
	Outside Flood Zone	Inside Flood Zone	*p*-Value	Outside Flood Zone	Inside Flood Zone	*p*-Value
Hispanic Immigrant ^1^	71.0	29.0	0.000	50.0	50.0	0.522
US-Born Hispanic ^1^	91.1	8.9	0.330	58.1	41.9	0.235
Non-Hispanic Black ^1^	87.1	12.9	0.818	49.3	50.7	0.761
Non-Hispanic Other Minority ^1^	61.1	38.9	0.002	70.0	30.0	0.138
Non-Hispanic White ^1^	90.2	9.8	0.002	44.3	55.7	0.099
SES ^2^	−0.0	0.0	0.776	−0.1	0.1	0.059
Renter ^1^	83.0	17.0	0.326	50.4	49.6	0.505
Proximity to Coast/Beach ^2^	2.5	2.2	0.081	2.2	2.4	0.114
Proximity to River/Lake ^2^	2.1	1.9	0.427	2.2	2.2	0.623
Flood Mitigation Composite ^2^	3.8	3.1	0.022	4.0	3.1	0.714
NFIP Contents Insurance ^1^	84.3	15.7	0.148	37.4	62.2	0.000
Risk Perception Factor ^2^	0.0	−0.2	0.099	-0.0	0.0	0.668

^1^ Pearson chi-square was used to test the significance of differences in proportions; percentages are reported; ^2^ Mann-Whitney U test was used to test the significance of differences in mean ranks; mean values rather than ranks for each of the groups are reported for descriptive purposes.

**Table 4:** Generalized estimating equations: Pooled results for models predicting 100-year flood zone exposure.

Variable	Houston			Miami		
	Exp(B)	SE	95% CI ^1^	Exp(B)	SE	95% CI ^1^
US-Born Hispanic	0.326	0.457	−1.443–0.460 **	0.783	0.521	−1.268–0.778
Non-Hispanic Black	0.472	0.327	−1.395–0.107 **	0.940	0.318	−0.686–0.562
Non-Hispanic Other Minority	1.365	0.319	−0.323–0.945	0.466	0.583	−1.921–0.395
Non-Hispanic White	0.248	0.343	−2.069–0.719 ***	1.725	0.164	0.223–0.867 ***
SES Factor	1.169	0.146	−0.131–0.442	1.235	0.021	0.169–0.253 ***
Renter	0.793	0.182	−0.192–0.522	1.234	0.184	−0.183–0.540
Proximity to Coast/Beach	0.874	0.116	−0.364–0.095	1.089	0.057	−0.027–0.198
Proximity to River/Lake	1.039	0.125	−0.207–0.285	1.044	0.126	−0.203–0.289
Flood Mitigation Composite	0.678	0.094	−0.573–0.205 ***	0.983	0.098	−0.208–0.175
NFIP Contents Insurance	1.527	0.222	−0.013–0.859	1.984	0.074	0.538–0.832 ***
Risk Perception Factor	0.809	0.086	−0.380–0.044 **	0.956	0.100	−0.242–0.151

^1^ ** *p* < 0.05; *** *p* < 0.01; Notes: Hispanic immigrants are the reference group for the other race/ethnicity/nativity categories. Participant sex was included as a control variable and has a statistically non-significant relationship with flood risk in both MSAs.
